# Botanical Authenticity of Miraruira Sold in the Amazonas State, Brazil, Based on Chemical Profiling Using DI-MS and Chemometric Analyses

**DOI:** 10.3390/plants14132012

**Published:** 2025-07-01

**Authors:** Shelson M. da R. Braga, Felipe M. A. da Silva, Giovana A. Bataglion, Marcia G. A. de Almeida, Larissa O. de Souza, Rebeca dos S. França, Cesar A. S. de Souza, Francinaldo A. da Silva-Filho, Afonso D. L. de Souza, Hector H. F. Koolen, Maria L. B. Pinheiro

**Affiliations:** 1 Programa de Pós-Graduação em Química, Universidade Federal do Amazonas, Manaus 69080-900, AM, Brazil; shelson.fq@gmail.com (S.M.d.R.B.); felipemourams@gmail.com (F.M.A.d.S.); giovanabataglion@ufam.edu.br (G.A.B.); marciagrace19@gmail.com (M.G.A.d.A.); rdss.franca@gmail.com (R.d.S.F.); augusto.silva21@gmail.com (C.A.S.d.S.); souzadq@ufam.edu.br (A.D.L.d.S.); hkoolen@uea.edu.br (H.H.F.K.); 2 Centro de Apoio Multidisciplinar, Universidade Federal do Amazonas, Manaus 69080-900, AM, Brazil; lari65519@gmail.com (L.O.d.S.); francinaldoasf@gmail.com (F.A.d.S.-F.); 3 Departamento de Química, Universidade Federal do Amazonas, Manaus 69080-900, AM, Brazil; 4 Escola Superior de Ciências da Saúde, Universidade do Estado do Amazonas, Manaus 69065-130, AM, Brazil

**Keywords:** benzoquinones, *Connarus ruber*, diabetes, embelin, rapanone, suberonone

## Abstract

Miraruira is a medicinal plant-based product (MPBP) that is widely used in the state of Amazonas for the treatment of diabetes, though its botanical identity remains unclear, which raises concerns about authenticity and therapeutic consistency. One solution to this problem is the use of mass spectrometry-based approaches, which have emerged as powerful tools for verifying botanical origin based on chemical composition. Thus, to confirm the botanical authenticity of miraruira, direct-injection mass spectrometry (DI-MS) and chemometric analyses (PCA and HCA) were conducted on methanol fractions of *Salacia impressifolia* and *Connarus ruber*, both suspected sources of miraruira, as well as commercial samples obtained in street markets in Manaus, Brazil. Additionally, the hexane extracts of *C. ruber* and the commercial samples were screened for benzoquinones using DI-MS, as these compounds are recurrent in the genus *Connarus*. The DI-MS and PCA analyses revealed distinct chemical profiles for each species, and identified mangiferin and epicatechin as chemical markers for *S. impressifolia* and *C. ruber*, respectively. Furthermore, PCA demonstrated that all the commercial samples exhibited chemical profiles closely aligned with *C. ruber.* However, the HCA indicated variability among these samples, suggesting *C. ruber* or related *Connarus* species are the primary sources of miraruira. Moreover, embelin, rapanone, and suberonone were identified as the main compounds in the hexane extracts of *C. ruber* and the commercial products. This study successfully confirmed the botanical authenticity of miraruira, identified key bioactive compounds related to its traditional use in the treatment of diabetes symptoms, and demonstrated the effectiveness of DI-MS as a valuable tool for addressing authenticity issues in MPBPs.

## 1. Introduction

Miraruira, also regionally known as cipó-miraruira, is a traditional medicinal plant-based product (MPBP) that is widely used in the Amazon region of Brazil, particularly in the state of Amazonas, for the treatment of diabetes and other metabolic disorders. This MPBP is commonly sold in local markets and street fairs in the form of dried bark that is stored in labeled plastic bags. These labels may include basic information such as therapeutic indications, preparation methods, dosage and administration instructions, and its botanical name. Despite its widespread and longstanding use, there is a lack of phytochemical data and considerable ambiguity regarding its true botanical origin. Locally, the name “miraruira” is often associated with two distinct species: *Salacia impressifolia* (Celastraceae) and *Connarus ruber* (Connaraceae). Although this MPBP often lists *S. impressifolia* on the label, both species have been cited in the ethnobotanical and pharmacological literature [1,2]. Interestingly, pharmacological studies on *S. impressifolia* have primarily focused on the anticancer activity of quinonemethide triterpenoids [1], although other triterpenoid classes, such as friedelane, lupane, oleanane, and ursane, have also been reported in this species [3]. In contrast, pharmacological studies on *C. ruber* have investigated the in vitro and in vivo antiglycation effects of its aqueous extract, demonstrating promising activity for preventing or slowing the progression of various pathological conditions, including diabetes [2]. To date, only one study has examined its chemical composition, highlighting epicatechin as a major constituent of its aqueous extract [2,4].

The taxonomic uncertainty surrounding miraruira presents a significant challenge to the validation of its chemical identity, therapeutic efficacy, and its safety. Given the growing global interest in MPMPs for the management of chronic diseases such as diabetes [5,6], the clarification of miraruira’s botanical source and chemical composition is not only of regional importance, but it also contributes to the broader efforts of integrating traditional medicines into evidence-based healthcare frameworks. Botanical authenticity is a critical factor in the control, efficacy, and safety of medicinal plants and MPBPs. As alternative and traditional medicines gain popularity, the commercialization of MPBPs has become a significant economic activity in the Amazon region, where a wide variety of products are readily available in markets and street fairs [7,8]. However, one of the main challenges in this trade, particularly in the state of Amazonas, is the scarcity of reliable data concerning the identity and composition of these products. This lack of standardization and traceability raises serious concerns since the substitution or misidentification of plant species can result in reduced therapeutic efficacy or can even pose health risks to consumers [9,10]. Ensuring botanical authenticity is therefore essential in order to confirm that the product contains the correct bioactive compounds. In this context, chemical profiling, particularly through mass spectrometry-based (MS) techniques, has emerged as a powerful tool for the quality control and authentication of plant species and their derivatives. These analytical approaches enable the identification of chemical markers, which supports regulatory efforts and the safe integration of traditional products into broader healthcare systems [11].

Mass spectrometry-based analyses allow for the differentiation between botanical species, the detection of key chemical markers, and the identification of potential adulterations in complex plant matrices [11]. While electrospray ionization (ESI) is commonly employed in these studies due to its suitability for analysis of polar and ionic compounds, atmospheric pressure chemical ionization (APCI) has also gained prominence. As a “soft” ionization technique, APCI enables the analysis of both polar and non-polar molecules, thereby expanding the range of natural products that can be effectively characterized [11,12]. Additionally, APCI in negative ion mode may yield better responses than ESI for certain compound classes, such as flavonoids [13]. Moreover, the integration of MS techniques, particularly direct injection mass spectrometry (DI-MS), with chemometric tools such as principal component analysis (PCA) and hierarchical cluster analysis (HCA) has shown great promise. These statistical methods help uncover meaningful patterns in complex datasets, thus facilitating the identification of species-specific chemical profiles and the selection of robust molecular markers for verifying botanical authenticity [14].

In this context, the present study aims to examine the botanical authenticity of miraruira sold in the state of Amazonas, Brazil, based on the comparison of the chemical profile of *S. impressifolia*, *C. ruber,* and commercial samples, employing DI-MS and chemometric approaches (PCA and HCA). The results of this comparison should contribute to the enhancement of the knowledge regarding this MPBP and its therapeutic potential.

## 2. Results and Discussion

### 2.1. Clean-Up Evaluation Using HPLC-MS Analysis

**Figure 1:**
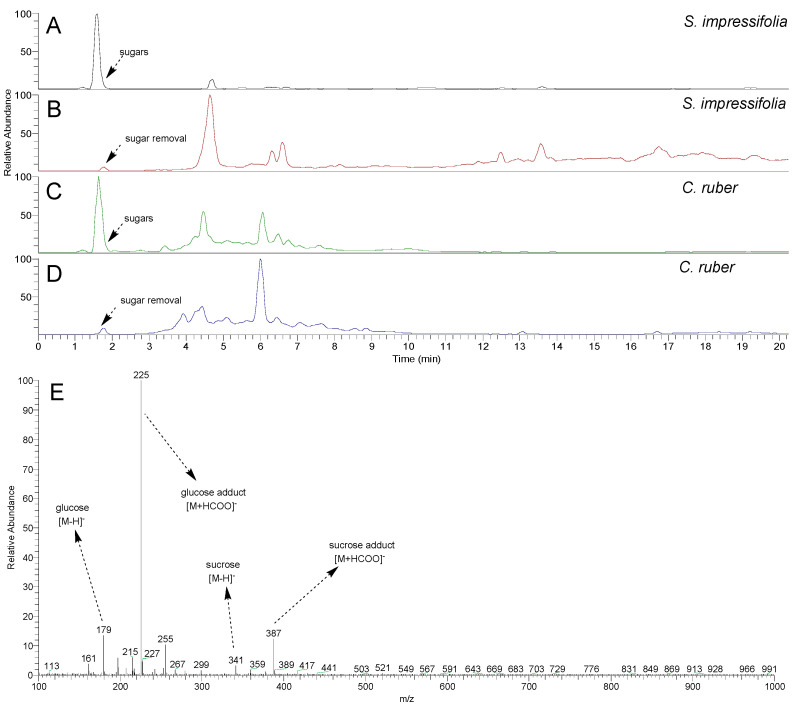
LC-MS chromatograms of the aqueous extracts of *S. impressifolia* (**A**) and *C. ruber* (**C**), and their respective methanol fractions (**B**,**D**) obtained using the SPE clean-up protocol. Mass spectrum corresponding to the peak, with a retention time of 1.60 min (**E**).

The LC-MS chromatograms of the aqueous extracts of *S. impressifolia* (Figure 1A) and *C. ruber* (Figure 1C) revealed the presence of major peaks, with a retention time of 1.60 **min** in both samples. Through the analysis of the full scan mass spectra of these peaks (Figure 1E), it was possible to observe the presence of characteristic ions of sugars, such as *m*/*z* 179 and 225 (glucose and adduct) and *m*/*z* 341 and 387 (sucrose and adduct), commonly found in plant matrices [15].

The methanol fractions of *S. impressifolia* (Figure 1B) and *C. ruber* (Figure 1D), obtained using the SPE clean-up protocol, showed a significant reduction in the chromatographic peak associated with sugars. This confirms the protocol’s efficiency in removing interfering substances that are detrimental to mass spectrometry analyses, thereby ensuring the suitability of these samples for DI-MS analysis. A comparative analysis also allowed us to verify that the clean-up effect was significantly greater for the aqueous extract of *S. impressifolia*, which showed a substantial signal gain after the removal of sugars.

### 2.2. Chemical Profile via DI-MS and Chemometric Analysis

**Figure 2:**
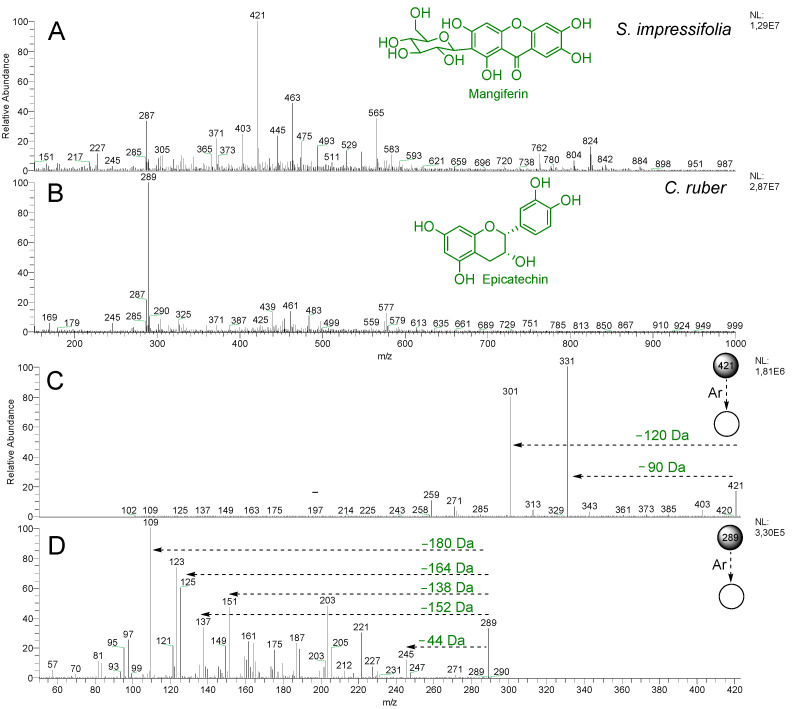
Mass spectra (negative mode) of the methanol fractions of *S. impressifolia* (**A**) and *C. ruber* (**B**), and MS/MS spectra of the ions at *m*/*z* 421 (**C**) and 289 (**D**).

The direct injection analysis of the methanol fractions of *S. impressifolia* and *C. ruber*, in the *m*/z range of 150–1000, revealed distinct profiles for both species. For *S. impressifolia*, the ion at *m*/*z* 421 was observed as the base peak (Figure 2A), while for *C. ruber*, the ion at *m*/*z* 289 was noted (Figure 2B). Several other ions of lower intensity were also observed in both samples. The commercial samples (A–J) exhibited similar chemical profiles among themselves, with the ion at *m*/*z* 289 as the base peak (Supporting Information—Figures S1–S10).

**Figure 3:**
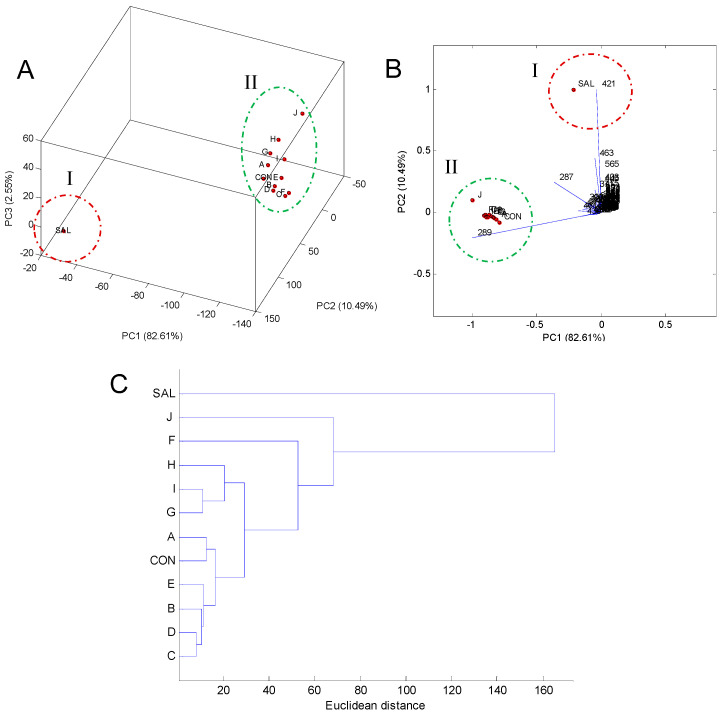
PCA score plots (**A**) and biplot (**B**) based on the chemical profile obtained using DI-MS of commercial samples (A–J) and samples of *S. impressifolia* (SAL) and *C. ruber* (CON), along with the HCA dendrogram (**C**).

In the PCA score plot (Figure 3A), generated from the relative abundance of the 114 ions that remained after the elimination of ions below 5%, the formation of two main groups was observed, with group I consisting of only the SAL sample (*S. impressifolia*) and group II comprising the CON sample (*C. ruber*) and the respective commercial samples (A-J). According to the PCA biplot (Figure 3B), the ions at *m*/*z* 289 and 421 were the main contributors to the segregation between groups I and II. Therefore, their MS/MS spectra were analyzed in an attempt to characterize these substances.

**Figure 4:**
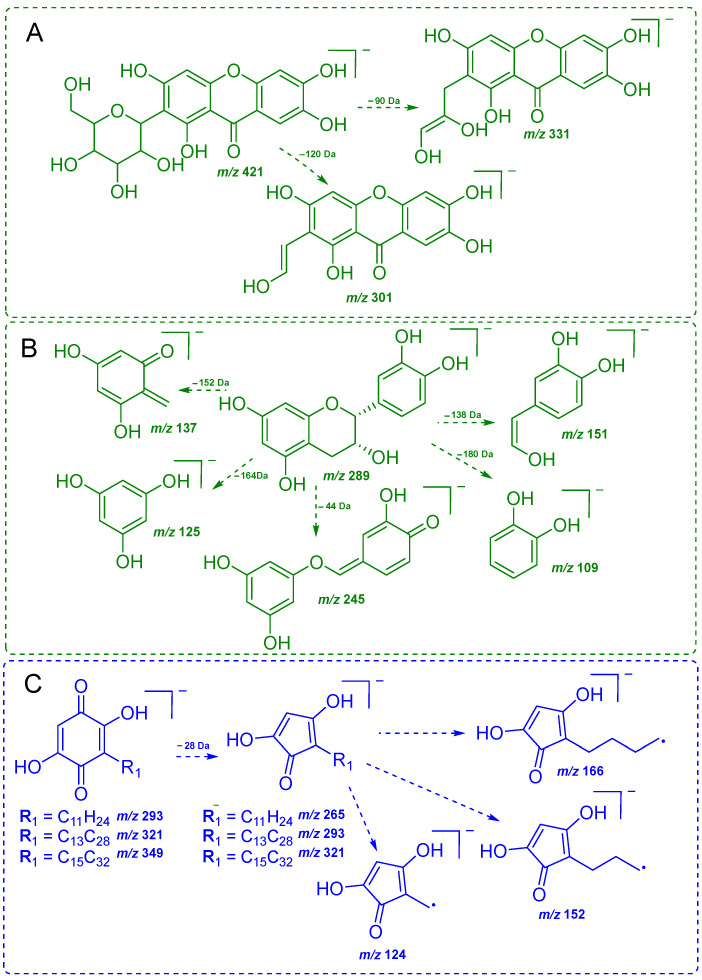
Proposed fragmentation pathways for mangiferin (*m*/*z* 421) (**A**), epicatechin (*m*/*z* 289) (**B**), and embelin (*m*/*z* 293), rapanone (*m*/*z* 321), and suberonone (*m*/*z* 349) (**C**).

The MS/MS spectrum of the ion at *m*/*z* 421, present in methanol fraction of *S. impressifolia* (Figure 2C), exhibited characteristic fragments of the xanthone mangiferin at *m*/*z* 403 (−18 Da, *m*/*z* 421 → *m*/*z* 403), *m*/*z* 331 (−90 Da, *m*/*z* 421 → *m*/*z* 331), *m*/*z* 301 (−120 Da, *m*/*z* 421 → *m*/*z* 301), *m*/*z* 271 (−150 Da, *m*/*z* 421 → *m*/*z* 271), and *m*/*z* 259 (−162 Da, *m*/*z* 421 → *m*/*z* 259) [16]. The neutral losses of 90 and 120 Da are in agreement with a *C*-hexoside compound (Figure 4A). Mangiferin was previously reported in several species of *Salacia*, such as *S. chinensis*, *S. oblonga*, *S. roxburghii*, and *S. reticulata* [17,18,19,20], but has not yet been reported in *S. impressifolia*. On the other hand, the MS/MS spectrum of the ion at *m*/*z* 289, present in the methanol fraction of *C. ruber* (Figure 2D), presented characteristic fragments of the A and B rings of epicatechin at *m*/*z* 151 (−138 Da, *m*/*z* 289 → *m*/*z* 151), *m*/*z* 137 (−152 Da, *m*/*z* 289 → *m*/*z* 137), *m*/*z* 125 (−164 Da, *m*/*z* 289 → *m*/*z* 125), and *m*/*z* 109 (−180 Da, *m*/*z* 289 → *m*/*z* 109), along with the ion at *m*/*z* 245 (−44 Da, *m*/*z* 289 → *m*/*z* 245) [21,22] (Figure 4B). Therefore, the ion at *m*/*z* 289 was tentatively identified as the flavonoid epicatechin. The ion at *m*/*z* 289, which was detected in commercial samples (A–J), exhibited similar MS/MS spectra. Epicatechin has been previously described as a major constituent of the aqueous extract of *C. ruber* and has also been noted as being promising for the treatment of various diseases, including diabetes, due to its potential to reduce oxidative stress, which is also related to its antioxidant properties [23,24,25].

Through the analysis of the HCA dendrogram (Figure 3C), it was possible to observe the formation of subgroups within group II, suggesting the existence of chemical variability among the commercial samples. The commercial samples B, C, D, and E exhibited strong chemical similarity among themselves, based on the chemical profile obtained by DI-MS, as well as the samples A and CON (*C. ruber*). These subgroups ended up connecting due to the greater similarity among themselves than with the subgroup containing samples G, H, and I. In turn, samples F and J showed the least chemical similarity among the commercial samples. This chemical variability may be due to the commercialization of miraruira based on other species of the *Connarus* genus, such as *C. perrottetii*, which also has a significant occurrence in the Amazon region [26].

### 2.3. Screening of Benzoquinones in C. ruber and Commercial Samples of Miraruira

**Figure 5:**
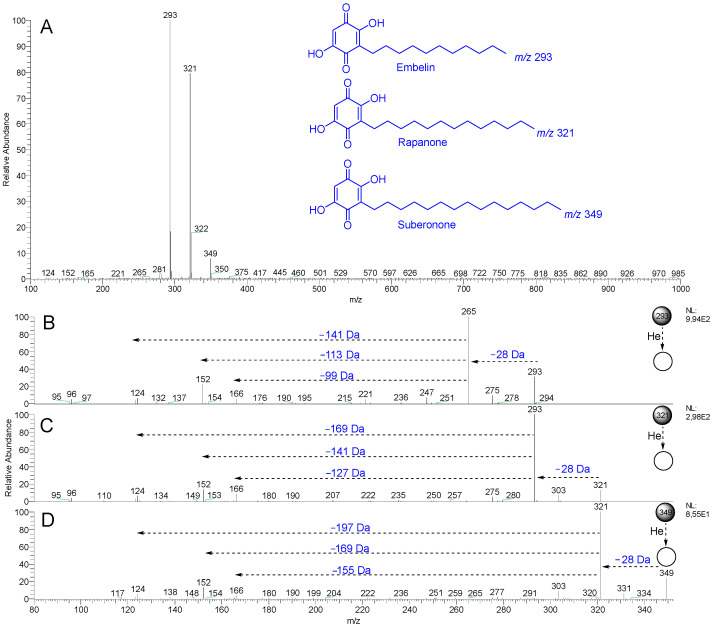
DI-MS spectrum (negative mode) of the hexane extract of *C. ruber* (**A**) and MS/MS mass spectra of the ions at *m*/*z* 293 (**B**), 321 (**C**), and 349 (**D**).

The DI-MS spectrum in the negative mode of the hexane extract of *C. ruber* revealed a base peak at *m*/*z* 293, another intense ion at *m*/*z* 321, and a minor ion at *m*/*z* 349 (Figure 5A). The commercial samples (A–J) also exhibited similar chemical profiles (Supporting Information—Figures S11–S20). The MS/MS spectra of these ions (Figure 5B–D) showed an initial loss of 28 Da (*m*/*z* 293 → 265; *m*/*z* 321 → 293; *m*/*z* 349 → 321), as well as common fragment ions at *m*/*z* 166, 152, 124, and 96 (Figure 4C), resulting from radical losses, all consistent with the benzoquinones embelin, rapanone, and suberonone [27]. The ions at *m*/*z* 293, 321, and 349 detected in the commercial samples (A–J) exhibited similar MS/MS spectra. A comparable benzoquinone profile has also been reported in the root wood ethyl acetate extract of *C. suberosus* [27]. Taken together, these observations reinforce the hypothesis that *C. ruber* or *Connarus* species are the main botanical sources of miraruira sold in the state of Amazonas.

Embelin, the major compound from the hexane extract of *C. ruber* and the commercial samples, has been exhaustively described as promising for the treatment of diabetes, due to its ability to significantly reduce blood glucose levels, improve insulin sensitivity, enhance antioxidant enzyme activity, and restore the normal histoarchitecture of pancreatic tissues in diabetic models. Additionally, embelin exhibits anti-inflammatory properties and can modulate lipid profiles, making it a potential adjuvant therapy alongside conventional antidiabetic medications [28,29,30]. Therefore, for miraruira, embelin along with epicatechin may play a crucial role in the treatment of diabetes symptoms, which justifies their traditional use.

### 2.4. Quality Control Challenges in the Commercialization of MPBPs: Lessons from the Case of Miraruira

The findings of this study underscore a critical and often overlooked issue in the sale of MPBPs: the botanical identity of the marketed material is frequently uncertain or wrongly assumed, rather than rigorously verified. In the case of miraruira, our analyses revealed that the species found in commercial products did not match the botanical identity labeled in some products and sometimes associated with this name [1]. This mismatch highlights a broader problem; the botanical nature of many MPBPs is not clearly established or authenticated prior to commercialization. This situation is not isolated, as it reflects a structural weakness in the quality control and traceability of herbal products, even those that are labeled and presumed to be regulated.

In a previous study, only 50.2% of samples acquired in markets matched the species described in the first edition of the Brazilian Pharmacopoeia [8]. The remainder consisted of substitutes or entirely different species, regardless of the geographic proximity to the native habitats of the original plants. Such practices not only compromise the therapeutic efficacy and safety of consumers but also threaten the conservation of both native and substitute species, which are often harvested without technical or environmental criteria. The adulteration and lack of standardization of MPBPs is a widespread issue, with particularly acute consequences in countries like Brazil, where the enforcement of quality control remains incipient [9]. The absence of robust methodologies for chemical analysis, such as the ones used in this study, contributes to the circulation of products with uncertain and/or potentially toxic compositions.

This broader pattern of adulteration is not limited to solid plant materials. Liquid extracts and resins, which are also widely used in phytotherapy and cosmetics, are similarly affected. A recent study on copaiba oil-resin, a product derived from *Copaifera* spp. from the Amazon, revealed that 75% of commercial samples were adulterated with triacylglycerides (e.g., soybean oil) or mineral oil [31]. Using NMR analysis combined with chemometric analysis, the authors detected significant levels of adulterants, often exceeding 90% of the sample composition. This finding illustrates that even high-value Amazonian products, traditionally perceived as natural and authentic, are subject to severe quality control failures—which emphasizes the need for analytical tools that can detect not only species substitution but also chemical adulteration.

The Brazilian regulatory framework, although undergoing changes, still shows a low representation of native species among licensed products. The new legislation, which distinguishes between herbal medicines and traditional herbal products, and which facilitates access to traditional knowledge, represents progress [32]. Nonetheless, implementation remains limited, and native species continue to be underrepresented among officially licensed products. These gaps allow for the continued commercialization of products with unknown or unverified botanical composition. The situation is particularly urgent in the Amazon region and its peripheral regions, where medicinal plants often represent the only accessible healthcare resource [33]. In such contexts, ensuring the authenticity and safety of MPBPs becomes not just a technical necessity but a public health imperative. Ethnobotanical surveys confirm the diversity and fluidity of species sold under common vernacular names in Amazonian markets [7]. While this reflects a rich cultural heritage, it also underscores the need for systematic monitoring, accurate taxonomic identification, and effective conservation strategies.

Ultimately, the data presented in this paper reinforce the notion that ensuring botanical authenticity in MPBPs is not merely about preventing adulteration; it is about recognizing that, in many cases, the botanical identity of what is sold has never been properly established to begin with. Addressing this issue requires coordinated action involving scientific validation, regulatory reform, and respect for traditional knowledge systems. Ensuring authenticity is not only vital for public health and consumer trust but also for the sustainable use and conservation of Brazil’s extraordinary botanical wealth.

## 3. Materials and Methods

### 3.1. General Experimental Procedures

HPLC-MS analysis was performed with an Accela HPLC system coupled to a TSQ Quantum Access (QqQ) mass spectrometer. DI-MS analyses were performed with a TSQ Quantum Access (QqQ) or with a LCQ Fleet mass spectrometer. The SPE procedures were performed using Pasteur pipettes as the support and KP-C18-HS (35–70 μm) (Biotage, Uppsala, Sweden) as the adsorbent. All the solvents used for extraction, chromatography, and MS experiments were of HPLC grade and were purchased from Tedia, and the water was purified using a Milli-Q system.

### 3.2. Plant Material

Commercial samples of miraruira were purchased from markets, fairs, and stores distributed throughout the city of Manaus, Amazonas, and were subsequently coded (A–J) and stored. Detailed information about each product is presented in Table 1. The plant material (bark of the trunk) from *Connarus ruber* (Poepp.) Planch. was collected in the city of Maués, Amazonas, from a previously identified individual. Its exsiccate was deposited in the herbarium of the Instituto Nacional de Pesquisas da Amazônia (INPA) under No. 230896. On the other hand, the plant material (bark of the trunk) from *Salacia impressifolia* (Miers) A.C. Sm. was collected in the Reserva Florestal Adolpho Ducke, in the city of Manaus, from a previously identified individual, with its exsiccate deposited in the INPA herbarium under No. 193803. Access to the commercial samples of miraruira, as well as to the plant material of the species *C. ruber*, was registered in the Sistema Nacional de Gestão do Patrimônio Genético e do Conhecimento Tradicional Associado (SisGen) under code A088AFE, while access to the plant material of *S. impressifolia* was previously registered in SisGen under code A715C2F.

### 3.3. Aqueous Extraction and Clean-Up Procedures

To obtain the aqueous extracts, approximately 1 g of each commercial sample, as well as bark of the trunk of *S. impressifolia* and *C. ruber*, previously ground in an analytical grinder model 80,374 (Hamilton Beach, SP, Brazil), was subjected to extraction with distilled water (50 mL) by decoction for 15 min. This procedure was adopted considering the instructions on the labels of some of the commercial miraruira samples, as well as previous experiences of our research group with the preparation of aqueous extracts from medicinal plants [34]. After the 15-min period, the extracts were filtered through Whatman 43 filter paper (Sigma Aldrich, MO, USA) and dried in the freeze dryer Alpha 1–2 LDplus (Martin Christ, Osterode am Harz, Germany). Once dried, all the extracts were properly weighed.

In order to remove interfering substances that are harmful to mass spectrometry analyses, such as salts and sugars, a clean-up procedure was performed on the extracts according to methodology previously reported [34]. Initially, 15 mg of each extract were solubilized in 1 mL of Milli-Q water and passed through a Pasteur pipette containing 300 mg of adsorbent (KP-C18-HS) previously activated with HPLC-grade MeOH (3 mL) and conditioned with Milli-Q water (5 mL). Subsequently, each column was washed with Milli-Q water (3 mL) (aqueous fraction) and then eluted with HPLC-grade MeOH (1 mL) into a 2 mL vial (methanol fraction). Finally, each methanol fraction was dried under a stream of nitrogen (N_2_) gas.

### 3.4. HPLC-MS and DI-MS Analyses

For the HPLC-MS analysis, the aqueous extracts and methanol fractions of *S. impressifolia* and *C. ruber* were prepared with a mixture of water–methanol (50:50, *v*/*v*) at a concentration of 1 mg/mL and injected (10 μL) into a Phenomenex Luna C18 (2) column (5 µm, 150 mm × 4.6 mm id) (Torrance, CA, USA). Milli-Q water (A) and MeOH (B) were used as the mobile phases. The following elution gradient was employed: 0–15 min, 20–80% B; 15–25 min, from 80% B at a flow rate of 1 mL/min. The analytical parameters used in mass spectrometry were as follows: discharge current: 5 µA; vaporizer temperature: 350 °C; capillary temperature: 250 °C; sheath gas: 35 psi; auxiliary gas: 15 arb; and mass range, *m*/*z* 150–1000.

For the DI-MS analysis, stock solutions (1 mg/mL) of the methanol fractions of the commercial samples (A-J) and authentic samples of *S. impressifolia* and *C. ruber* were prepared in HPLC-grade MeOH. Aliquots (10 µL) of the stock solutions were subsequently transferred to vials containing 1 mL of MeOH. Finally, 10 µL of the diluted solutions (10 ppm) were analyzed via direct injection (DI) into a triple quadrupole mass spectrometer TSQ Quantum Access, equipped with an APCI source operating in negative mode. The analytical parameters used were as follows: discharge current: 5 µA; vaporizer temperature: 350 °C; capillary temperature: 250 °C; sheath gas: 35 psi; auxiliary gas: 15 arb; and mass range, *m*/*z* 150–1000. The MS/MS spectra were obtained through collision-induced dissociation (CID) using argon as the collision gas and collision energy of 25 eV.

### 3.5. Chemometric Analysis

For the principal component analysis (PCA), at least 10 spectra from each sample were processed in the full scan mode, and then converted into an ion list containing absolute and relative intensity values for each ion in the *m*/*z* range from 150 to 1000. The relative intensity data for all ions were tabulated in Excel, and ions with an intensity below 5% relative to the most abundant ion were subsequently disregarded [14,35]. The resulting matrix was analyzed using the Chemoface software (version 1.5) [36]. In turn, a hierarchical clustering analysis (HCA) was carried out using the calculation of Euclidean distance and average linkage. The first four principal components were considered in the HCA analysis.

### 3.6. Screening of Benzoquinones in C. ruber and Commercial Samples of Miraruira

Since the benzoquinones rapanone, embelin, and suberonone are non-polar natural products recurrent in the genus *Connarus* [37,38], a screening based on non-polar extraction and DI-MS analysis was performed with *C. ruber* and all the commercial samples as follows: approximately 1 g of *C. ruber* and each commercial sample, previously ground, was subjected to extraction with hexane (50 mL) assisted by ultrasound for 15 min. After this period, the extracts were dried using rotary evaporation and then weighed.

For the DI-MS analysis, the stock solutions (1 mg/mL) of the hexane extracts from the *C. ruber* and the commercial samples of miraruira (A–J) were prepared in HPLC-grade ethyl acetate as previously described. Finally, 10 µL of the diluted solutions (10 ppm) were analyzed via DI into a mass spectrometer LCQ Fleet, equipped with an APCI source operating in the negative mode. The analytical parameters used were as follows: discharge current: 5 µA; vaporizer temperature: 350 °C; capillary temperature: 250 °C; sheath gas: 35 psi; auxiliary gas: 15 arb; and mass range: *m*/*z* 150–1000. The MS/MS spectra were obtained through collision-induced dissociation (CID) using helium as the collision gas and collision energy of 25%.

## 4. Conclusions

This study successfully demonstrated the utility of DI-MS combined with chemometric analyses as a reliable and efficient approach for confirming the botanical authenticity of miraruira, a medicinal plant-based product (MPBP) traditionally used in the treatment of diabetes. The observed chemical distinction between the analyzed samples of *S. impressifolia* and *C. ruber*, along with the identification of chemical markers such as mangiferin and epicatechin, highlights the potential of this approach for the authentication of MPBPs in the Amazon region. However, since the chemical composition of plants can vary depending on soil, climatic conditions, herbivory, and other environmental factors, studies involving multiple specimens per species may be necessary to validate these markers at the species level and ensure the success of this approach. Additionally, the identification of other key bioactive compounds like embelin, rapanone, and suberonone, further supports the therapeutic relevance of miraruira in managing symptoms associated with diabetes.

Although the consistent presence of *Connarus* species in commercial samples validates the traditional knowledge, it simultaneously exposes that the species found in commercial products did not match the botanical identity labeled in some products and sometimes associated with this name. These findings underscore a critical and often overlooked reality in the commercialization of MPBPs: the botanical identity of many marketed products is frequently uncertain, wrongly assumed, or never rigorously verified prior to commercialization. In a country like Brazil, where the biodiversity of medicinal plants is immense but regulatory oversight remains limited, this issue has serious implications for consumer safety, public health, and the preservation of biodiversity. The data presented in this paper reinforce the urgency of implementing coordinated actions related to the quality control of traded medicinal plants and MPMPs, especially regarding the botanical authenticity issues.

## Figures and Tables

**Table 1:** Origin of commercial miraruira samples and information available on their labels.

Samples	Origin	Label Information
A	Market	Therapeutic indication, preparation method, Dosage, and administration
B	Fair	Therapeutic indication, dosage, and administration
C	Emporium	No information
D	Emporium	Botanical name ^a^, therapeutic indication, preparation method, dosage, and administration
E	Emporium	Botanical name ^a^, therapeutic indication, preparation method, dosage, and administration
F	Fair	Botanical name ^a^, therapeutic indication, preparation method, dosage, and administration
G	Emporium	Preparation method, dosage, and administration
H	Emporium	Preparation method, dosage, and administration
I	Fair	No information
J	Market	No information

^a^ *S. impressifolia.*

## Data Availability

The original contributions presented in the study are included in the article; further inquiries can be directed to the corresponding author.

## Supplementary Materials

The following supporting information can be downloaded at: https://www.mdpi.com/article/10.3390/plants14132012/s1, Figure S1: Mass spectrum (negative mode) of the methanol fraction of commercial sample A; Figure S2: Mass spectrum (negative mode) of the methanol fraction of commercial sample B; Figure S3: Mass spectrum (negative mode) of the methanol fraction of commercial sample C; Figure S4: Mass spectrum (negative mode) of the methanol fraction of commercial sample D; Figure S5: Mass spectrum (negative mode) of the methanol fraction of commercial sample E; Figure S6: Mass spectrum (negative mode) of the methanol fraction of commercial sample F; Figure S7: Mass spectrum (negative mode) of the methanol fraction of commercial sample G; Figure S8: Mass spectrum (negative mode) of the methanol fraction of commercial sample H; Figure S9: Mass spectrum (negative mode) of the methanol fraction of commercial sample I; Figure S10: Mass spectrum (negative mode) of the methanol fraction of commercial sample J; Figure S11: Mass spectrum (negative mode) of the hexane extract of commercial sample A; Figure S12: Mass spectrum (negative mode) of the hexane extract of commercial sample B; Figure S13: Mass spectrum (negative mode) of the hexane extract of commercial sample C; Figure S14: Mass spectrum (negative mode) of the hexane extract of commercial sample D; Figure S15: Mass spectrum (negative mode) of the hexane extract of commercial sample E; Figure S16: Mass spectrum (negative mode) of the hexane extract of commercial sample F; Figure S17: Mass spectrum (negative mode) of the hexane extract of commercial sample G; Figure S18: Mass spectrum (negative mode) of the hexane extract of commercial sample H; Figure S19: Mass spectrum (negative mode) of the hexane extract of commercial sample I; Figure S20: Mass spectrum (negative mode) of the hexane extract of commercial sample J.

## Abbreviations

The following abbreviations are used in this manuscript:

MPBP	Medicinal Plant-Based Product
DI-MS	Direct-Injection Mass Spectrometry
PCA	Principal Component Analysis
HCA	Hierarchical Cluster Analysis
