# Supplementary material for: Botanical Authenticity of Miraruira Sold in the Amazonas State, Brazil, Based on Chemical Profiling Using DI-MS and Chemometric Analyses
Source: Plants (Basel). 2025 Jul 1;14(13):2012. doi: 10.3390/plants14132012 (PMC12251871; doi:10.3390/plants14132012)
Supplement: Supplementary file 1 [file plants-14-02012-s001.zip › plants-3694145-supplementary.pdf]

Supplementary materials

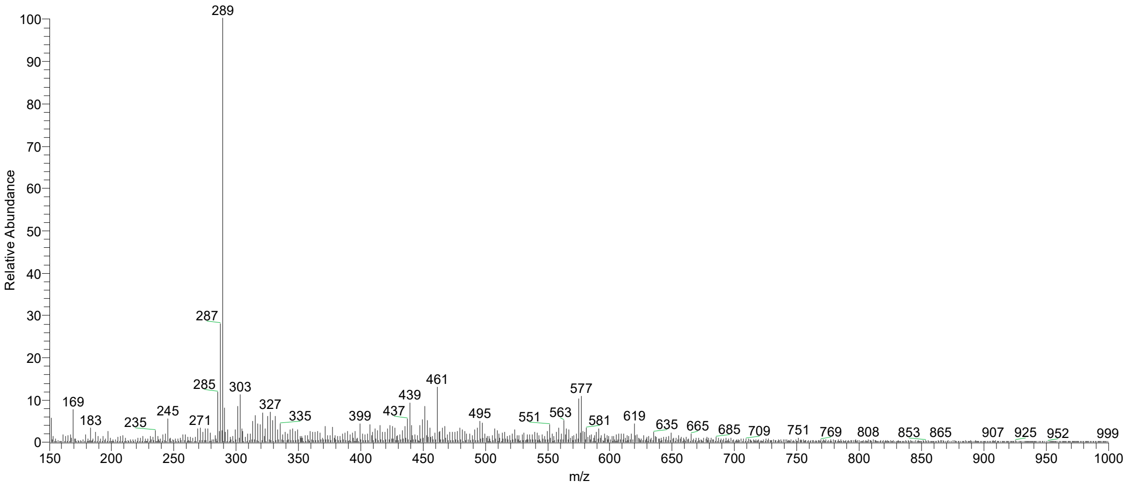

Figure S1. Mass spectrum (negative mode) of the methanol fraction of commercial sample A.

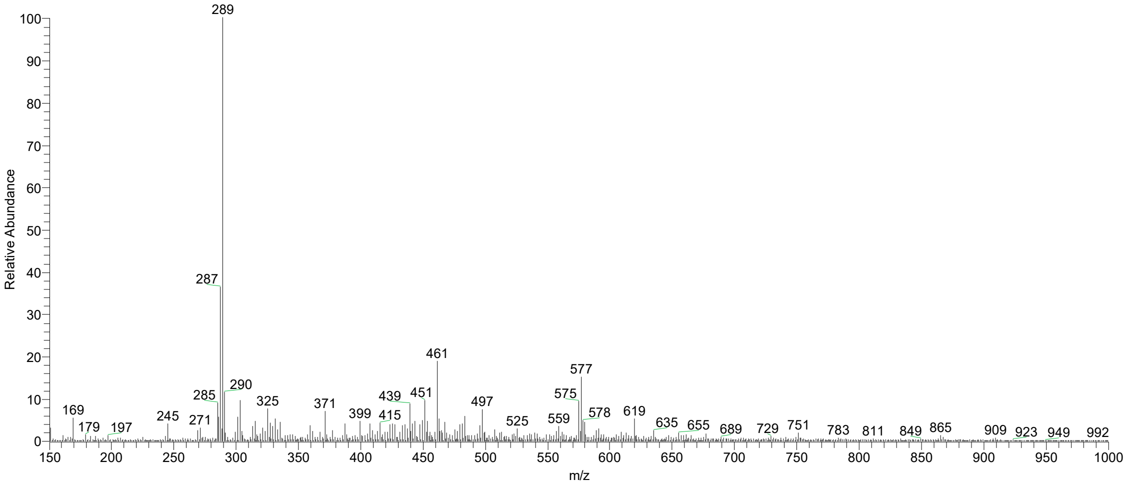

Figure S2. Mass spectrum (negative mode) of the methanol fraction of commercial sample B.

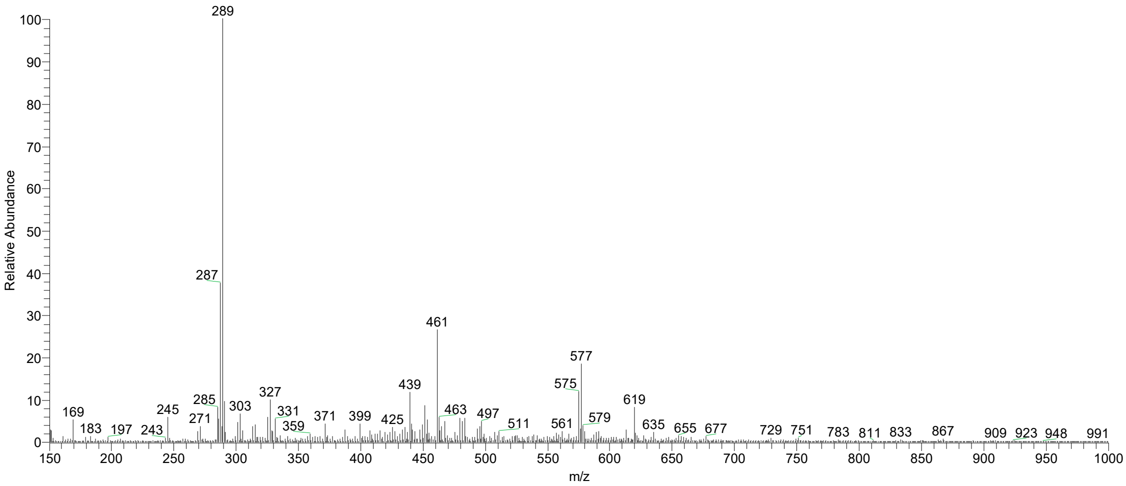

Figure S3. Mass spectrum (negative mode) of the methanol fraction of commercial sample C.

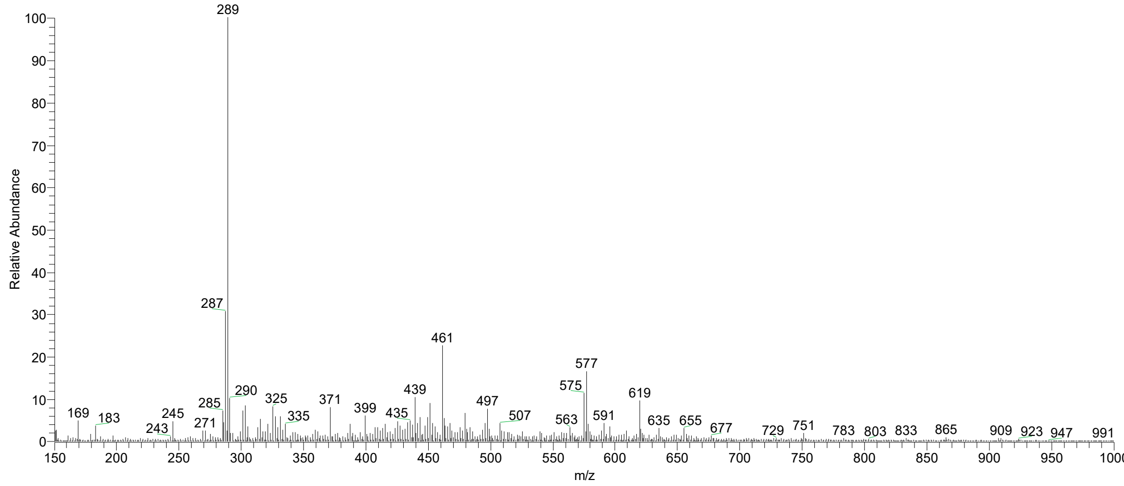

Figure S4. Mass spectrum (negative mode) of the methanol fraction of commercial sample D.

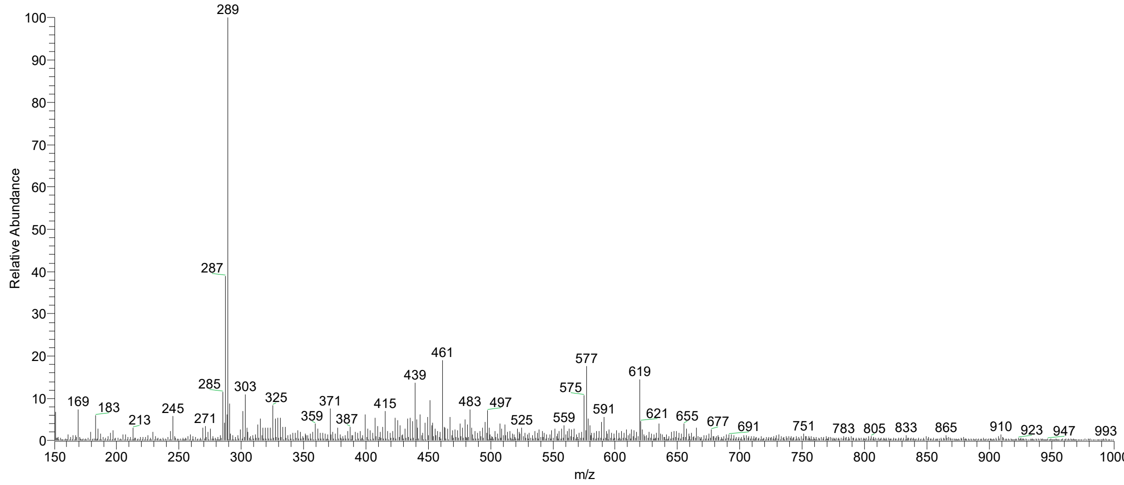

Figure S5. Mass spectrum (negative mode) of the methanol fraction of commercial sample E.

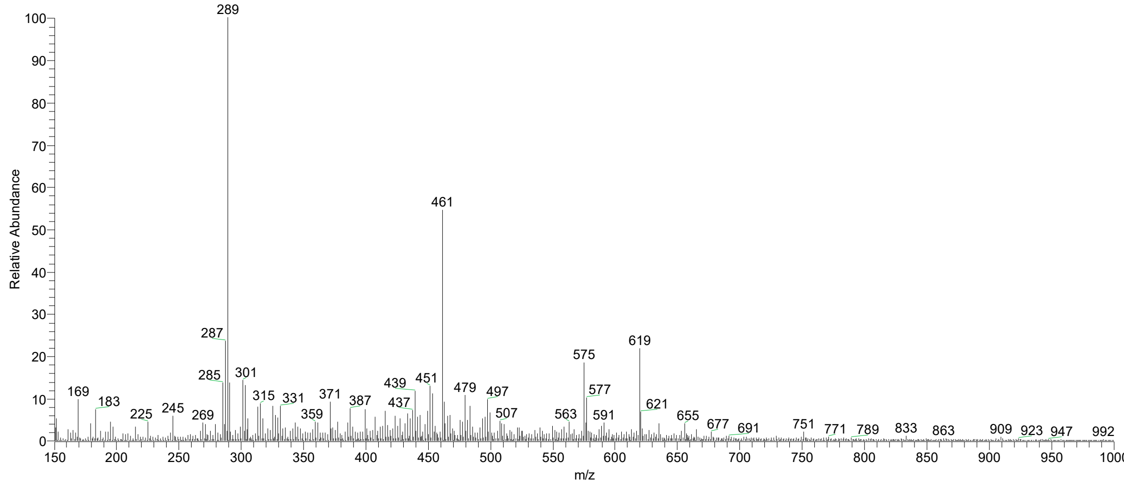

Figure S6. Mass spectrum (negative mode) of the methanol fraction of commercial sample F.

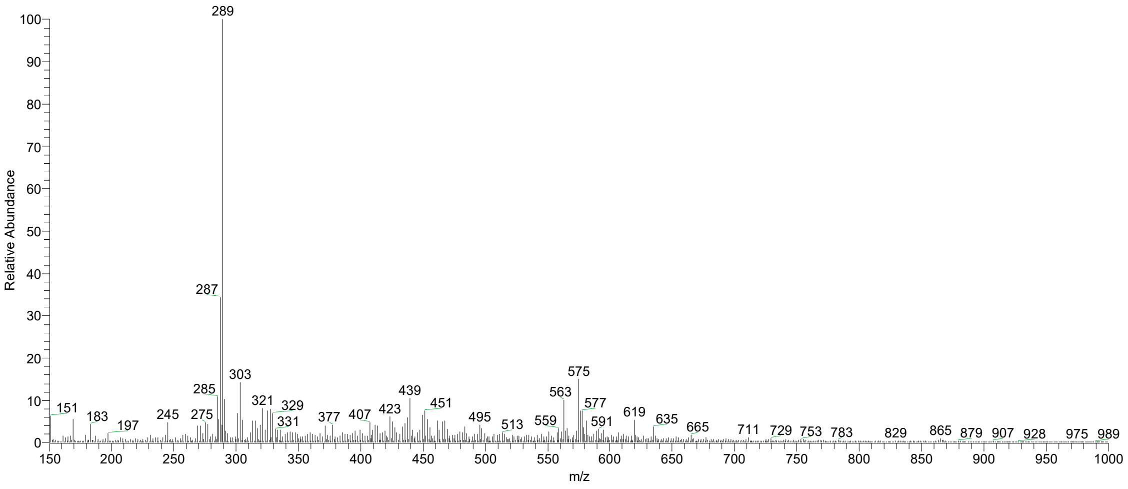

Figure S7. Mass spectrum (negative mode) of the methanol fraction of commercial sample G.

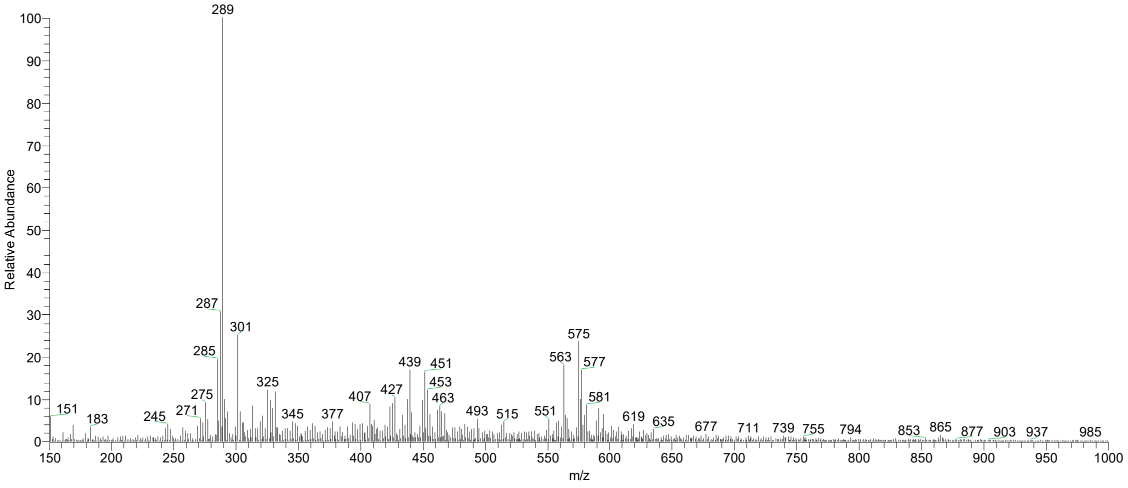

Figure S8. Mass spectrum (negative mode) of the methanol fraction of commercial sample H.

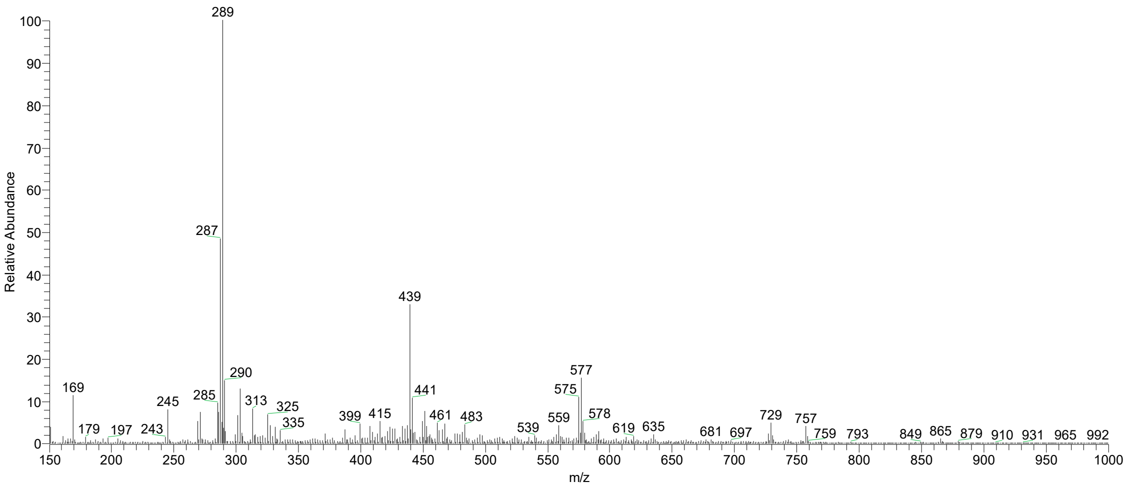

Figure S9. Mass spectrum (negative mode) of the methanol fraction of commercial sample I.

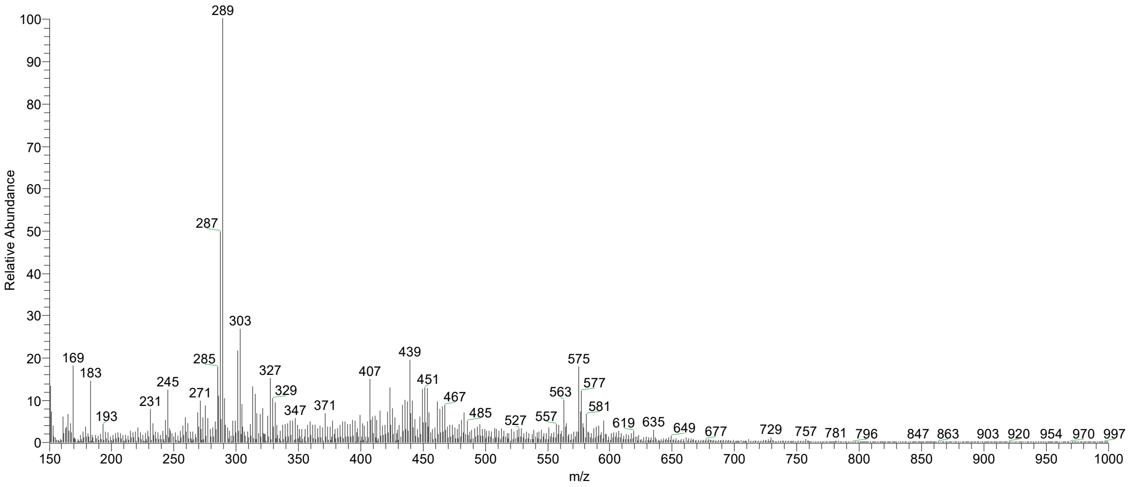

Figure S10. Mass spectrum (negative mode) of the methanol fraction of commercial sample J.

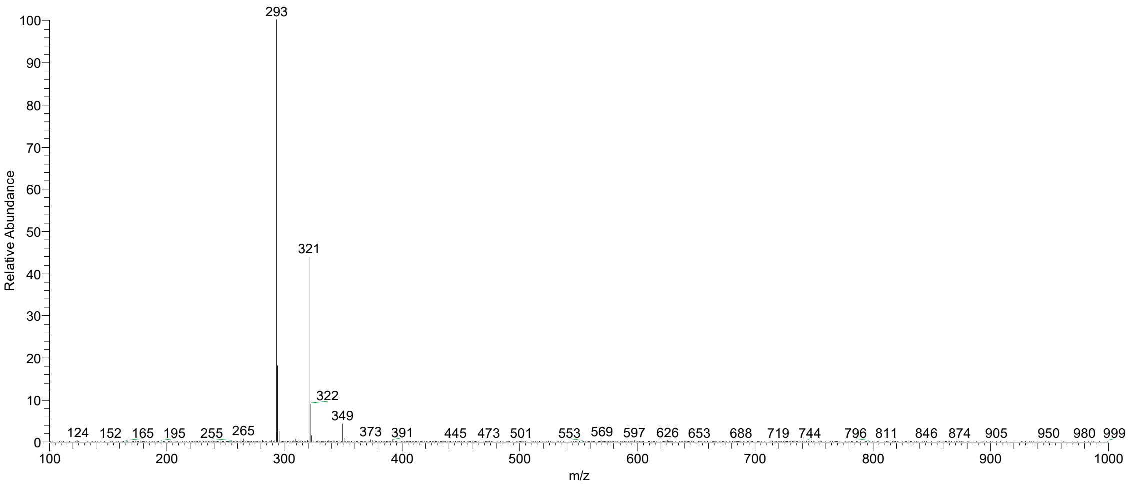

Figure S11. Mass spectrum (negative mode) of the hexane extract of commercial sample A.

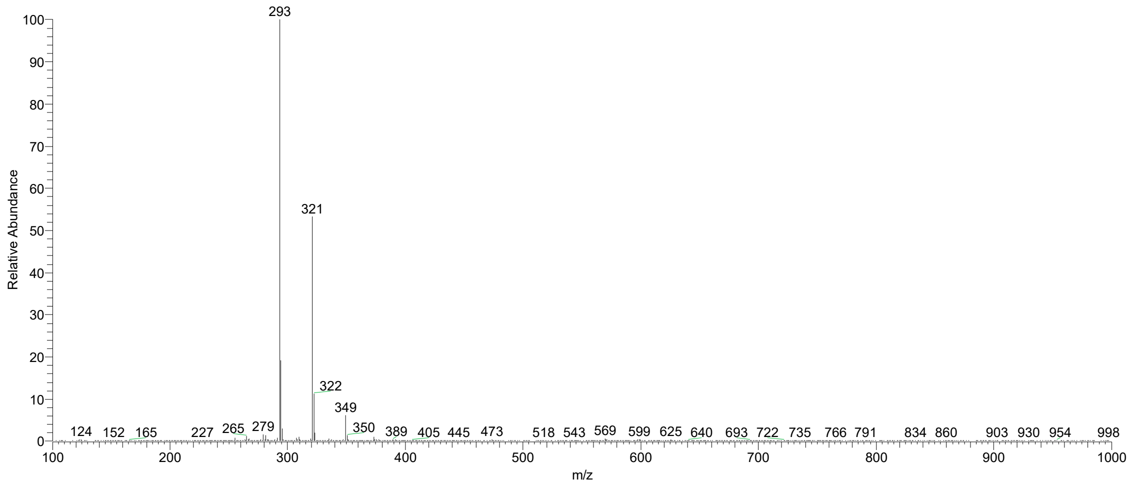

Figure S12. Mass spectrum (negative mode) of the hexane extract of commercial sample B.

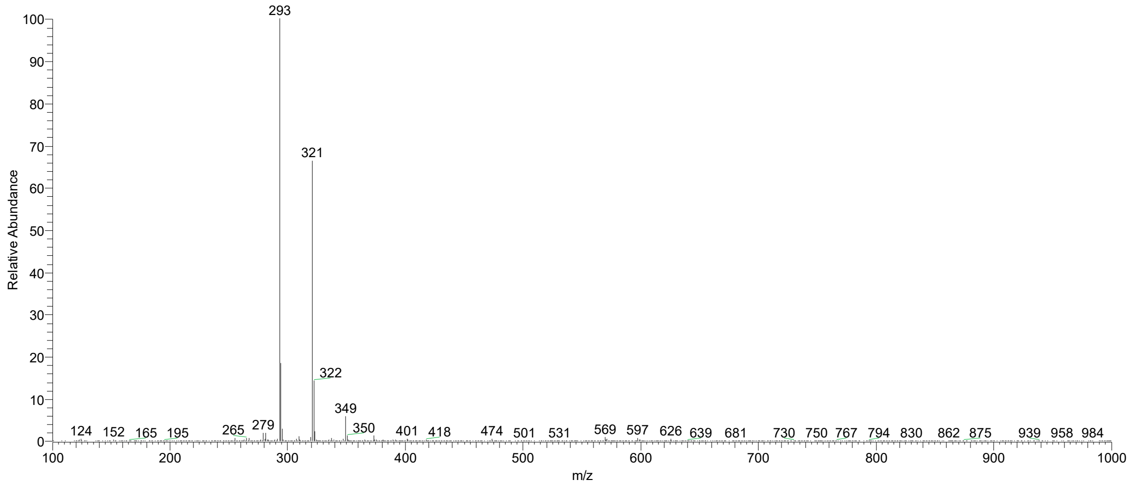

Figure S13. Mass spectrum (negative mode) of the hexane extract of commercial sample C.

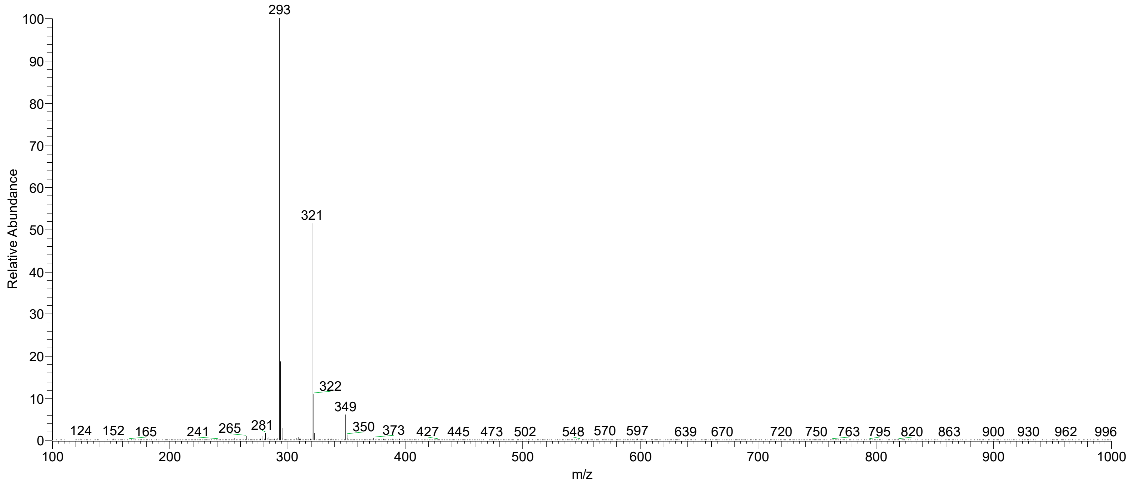

Figure S14. Mass spectrum (negative mode) of the hexane extract of commercial sample D.

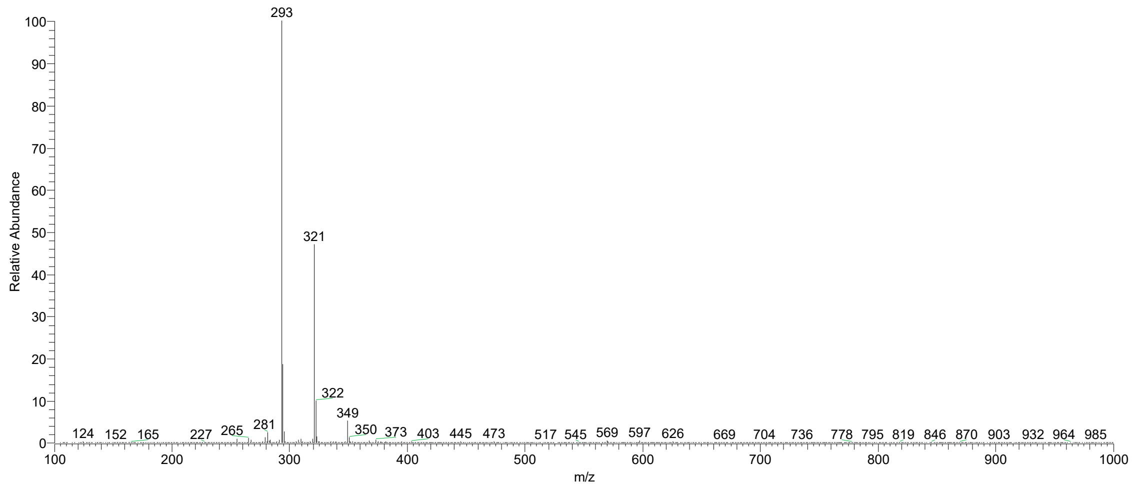

Figure S15. Mass spectrum (negative mode) of the hexane extract of commercial sample E.

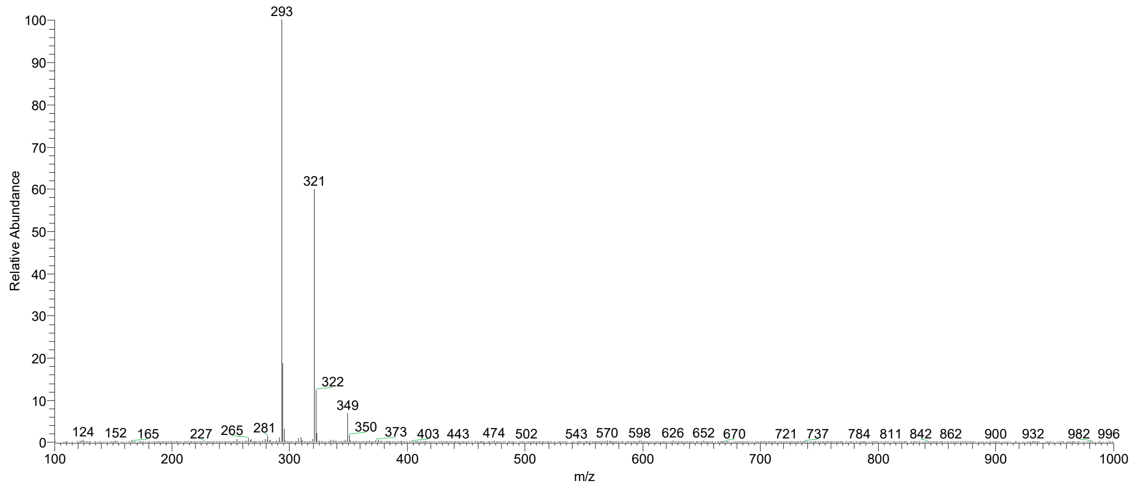

Figure S16. Mass spectrum (negative mode) of the hexane extract of commercial sample F.

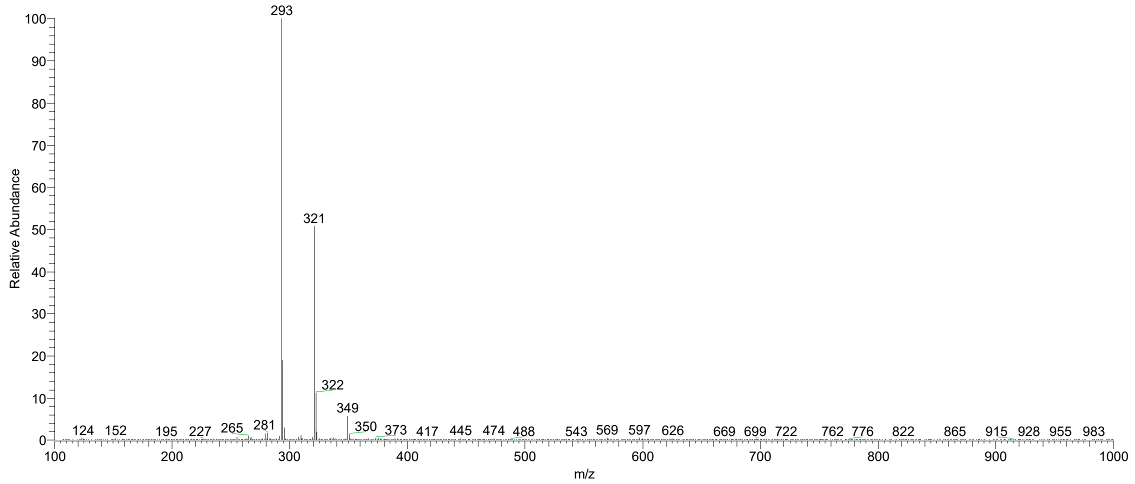

Figure S17. Mass spectrum (negative mode) of the hexane extract of commercial sample G.

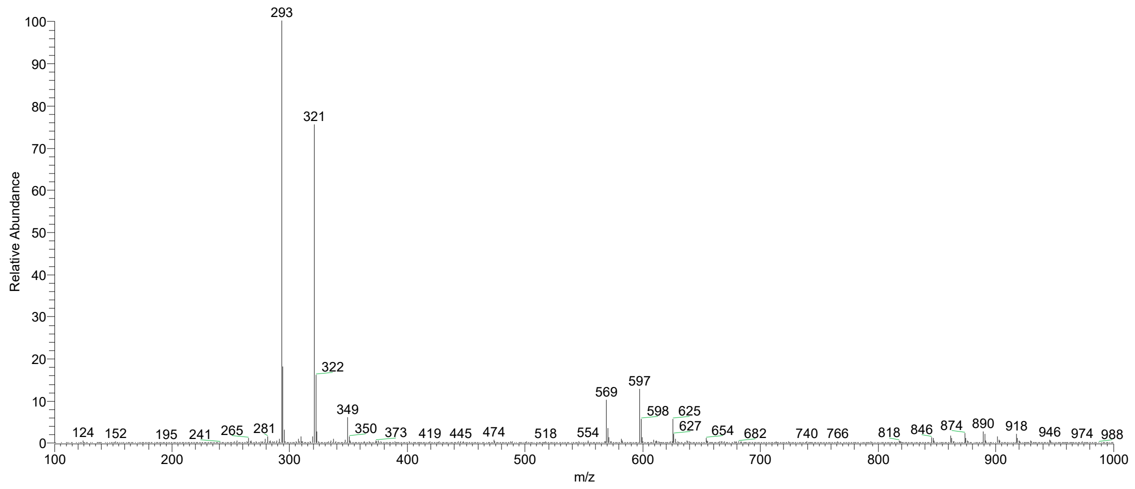

Figure S18. Mass spectrum (negative mode) of the hexane extract of commercial sample H.

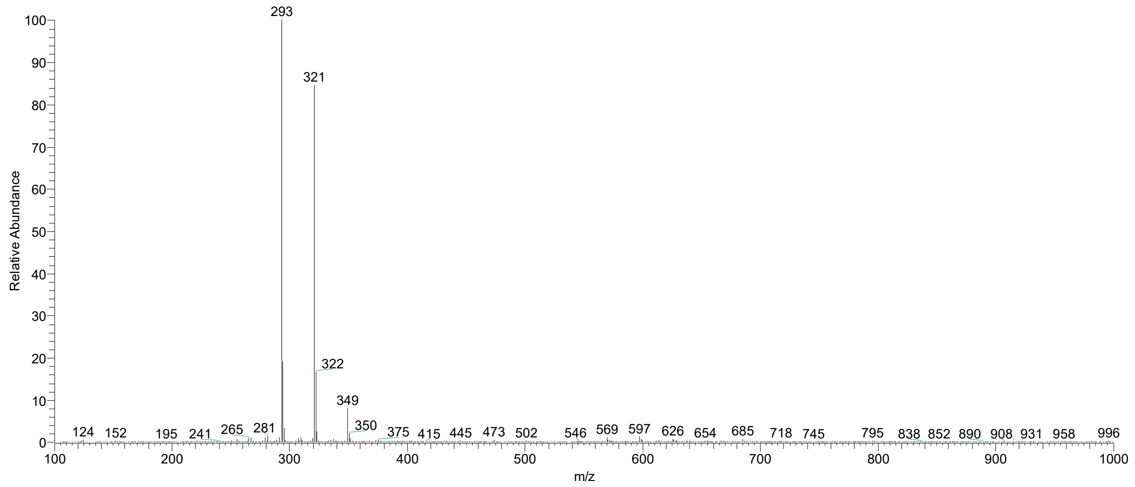

Figure S19. Mass spectrum (negative mode) of the hexane extract of commercial sample I.

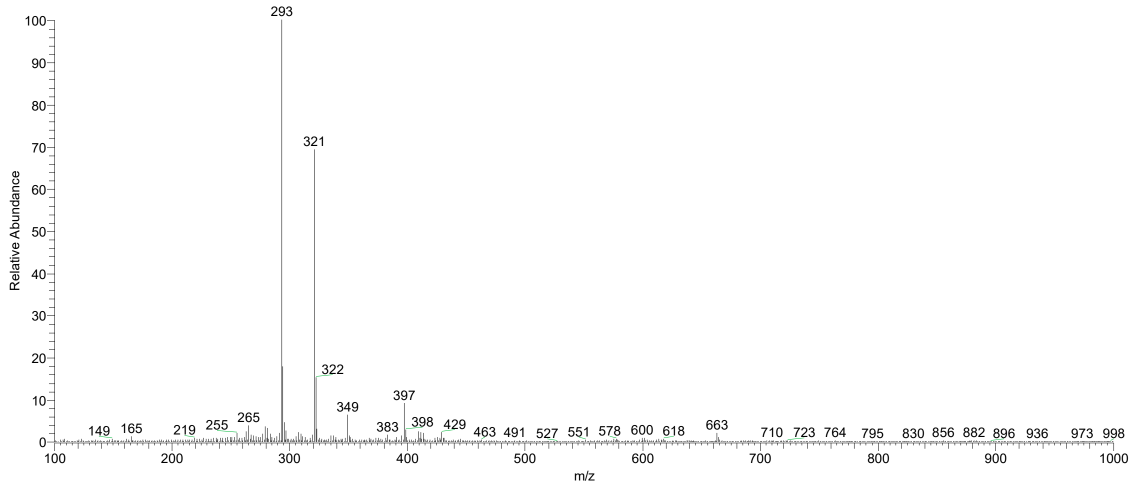

Figure S20. Mass spectrum (negative mode) of the hexane extract of commercial sample J.
